# Nonreplicative functions of the origin recognition complex

**DOI:** 10.1080/19491034.2018.1516484

**Published:** 2018-10-20

**Authors:** Varvara V. Popova, Alexander V. Brechalov, Sofia G. Georgieva, Daria V. Kopytova

**Affiliations:** Department of Transcription Regulation and Chromatin Dynamics, Institute of Gene Biology, Russian Academy of Sciences, Moscow, Russia

**Keywords:** Origin recognition complex, ORC, mRNP, mRNA export, TREX-2, SIR, HP1, cohesin, sister chromatids, centrosomes

## Abstract

Origin recognition complex (ORC), a heteromeric six-subunit complex, is the central component of the eukaryotic pre-replication complex. Recent data from yeast, frogs, flies and mammals present compelling evidence that ORC and its individual subunits have nonreplicative functions as well. The majority of these functions, such as heterochromatin formation, chromosome condensation, and segregation are dependent on ORC-DNA interactions. Furthermore, ORC is involved in the control of cell division via its participation in centrosome duplication and cytokinesis. Recent findings have also demonstrated a direct interaction between ORC and mRNPs and highlighted an essential role of ORC in mRNA nuclear export. Along with the growth of evolutionary complexity of organisms, ORC complex functions become more elaborate and new functions of the ORC sub-complexes and individual subunits have emerged.

## Introduction

Origin Recognition Complex (ORC) was first characterized in *Saccharomyces cerevisiae* as a complex of six subunits (ORC1–6) that binds to DNA replication origins [1]. Homologues for all six subunits constituting ORC were identified subsequently in other organisms, including mammals [2–5].

ORC plays a key role in the initiation of replication. It binds to the DNA replication origins in an ATP-dependent manner and serves as a scaffold for pre-replication complex (pre-RC) assembly [5]. ORC recruits other initiation proteins, such as Cdc6, Cdt1, and two heterohexameric Mcm2–7 helicase complexes to the origins during the G1 phase. Only the origins with properly assembled pre-RC may function as the replication initiation sites; pre-RC, thus serving as a licensing point for their replication competence. Such origin licensing provides the cell with a sort of accounting system which guarantees that every part of the genome is replicated only once.

Despite the fact that the components of eukaryotic DNA replication initiation are highly conserved, the organization and functions of the ORC subunits can differ significantly between organisms. In yeast, all six subunits of ORC remain bound to the chromatin throughout the cell cycle and ORC activity is regulated by phosphorylation [6]. In contrast, in metazoa, ORC has a more dynamic interaction with DNA. For example, the complex is not bound to the chromatin during the S and M phases in *Xenopus* [7] and human cells [8]. Interestingly, in *Drosophila* and human cells, the ORC1 subunit appears to be a key regulator of ORC replicative function. This subunit is degraded at the end of the M phase by the anaphase-promoting complex in *Drosophila* [9], while in human, it undergoes an ubiquitin-mediated proteolysis at the end of the G1/S transition [10]. However, in both organisms, ORC1 re-binding to chromatin is essential for the pre-RC assembly during G1 phase [7,8]. Moreover, in mammals the BAH (bromo-adjacent homology) domain of ORC1 recognizes the H4K20me2 modification [11], which is associated with replication. Besides that, human ORC2, 3, 4, and 5 form the core of the complex and are associated with each other throughout the entire cell cycle, while the smallest subunit, ORC6, interacts with the complex in a transient manner [12,13]. Taken together, multicellular organisms have more complex regulation of ORC that reflects the evolutionary emergence of new additional functions of the complex.

Although the ORC’s role in the initiation of DNA replication is the most thoroughly studied function of the complex, a large body of data has been accumulated so far, which demonstrates its involvement in other cellular processes. In the last few decades, involvement of ORC in numerous highly diverse cellular functions was discovered, e. g. the formation of heterochromatin, chromosome condensation and segregation, centrosome duplication, cytokinesis and mRNA nuclear export [14–17]. The sub-complexes or the individual subunits of ORC are localized not only in nuclei and on the mitotic chromosomes, but in the cytoplasm as well [18–21]. Interestingly, ORC subunits have also been detected in terminally differentiated mammalian cells that do not undergo subsequent cell divisions, for example, cardiomyocytes and neurons [22]. This fact points to the significance of the nonreplicative functions of ORC. This current review is devoted to the diverse nonreplicative functions of ORC in various organisms.

## Heterochromatin formation and transcription regulation

**Figure 1.**
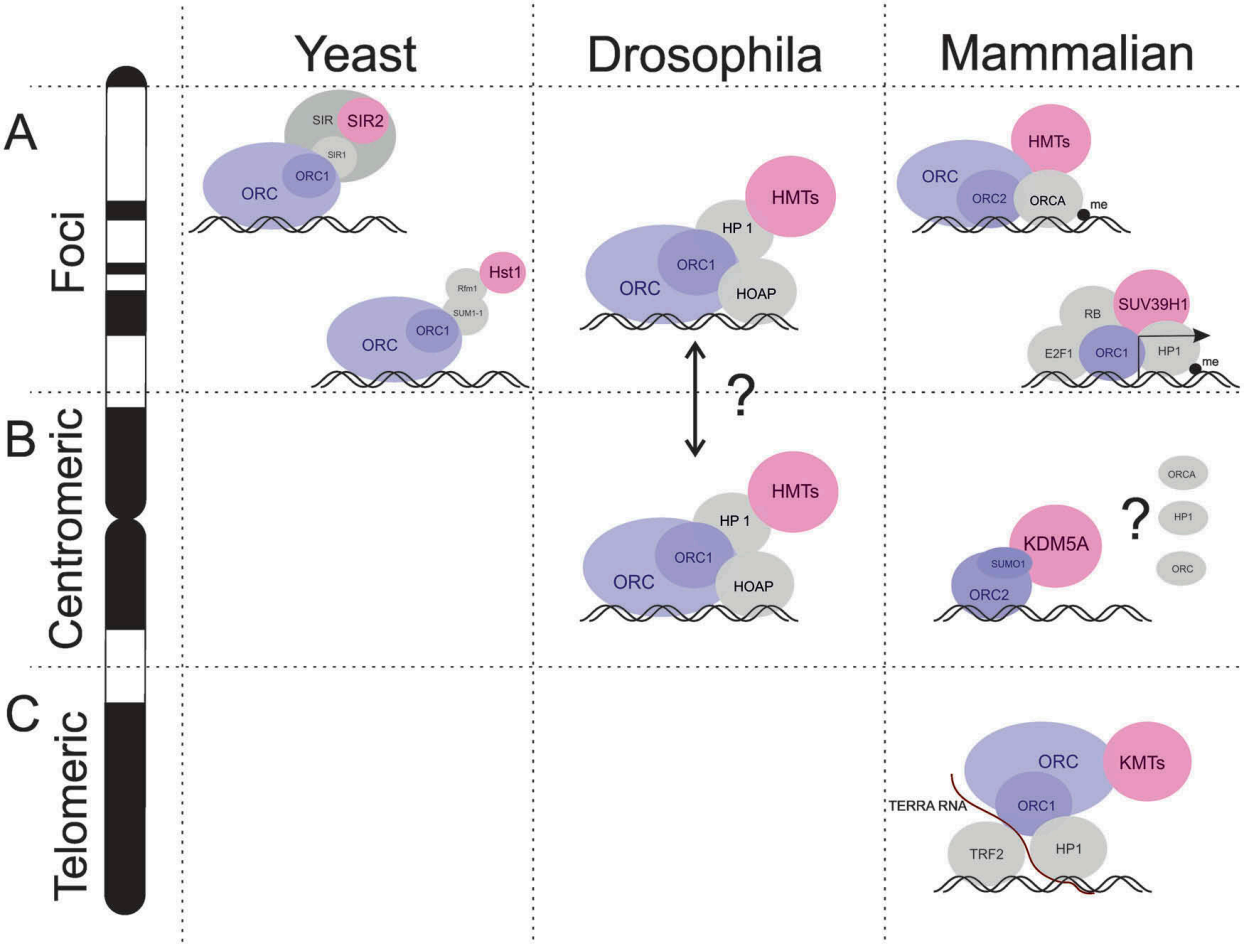
Chromatin related functions of ORC in different species. (a) ORC takes a part in transcriptional repression in yeasts, insects and mammals. Either with supplemental proteins or ORC complex alone recruits the histone-methyltransferase (HMT) to the genome locus or particular gene (in mammals, only). (b) In *Drosophila* and mammals, ORC were found in the centromeric region. In mammals, the presence of ORC subunits (besides ORC2) and other proteins (i. e. ORCA and HP1) remains unclear. (c) In mammals, ORC is reqruited to the telomeric regions in the RNA-dependent manner, via interaction with TRF2 and HP1 proteins (see detailed description in the text).

The first discovered nonreplicative function of ORC was an involvement in the establishment of the silent mating-type loci (HMR and HML) in *S. cerevisiae* [23–25]. The silent HML and HMR loci are flanked by the sequences to which Silent Information Regulator (SIR) proteins are recruited (Figure 1a) [26]. These histone-modifying proteins do not recognize specific DNA sequences by themselves. Therefore, the recruitment of the SIR proteins to the boundaries of the silent loci requires additional DNA-binding proteins, such as Abf1, Rap1, and ORC [26]. ORC1 plays a key role in this process (Figure 1a). ORC1 interacts with the SIR1 protein and recruits the remaining components of the SIR complex including histone deacetylase SIR2 to the chromatin [7,27–30].

**Figure 2.**
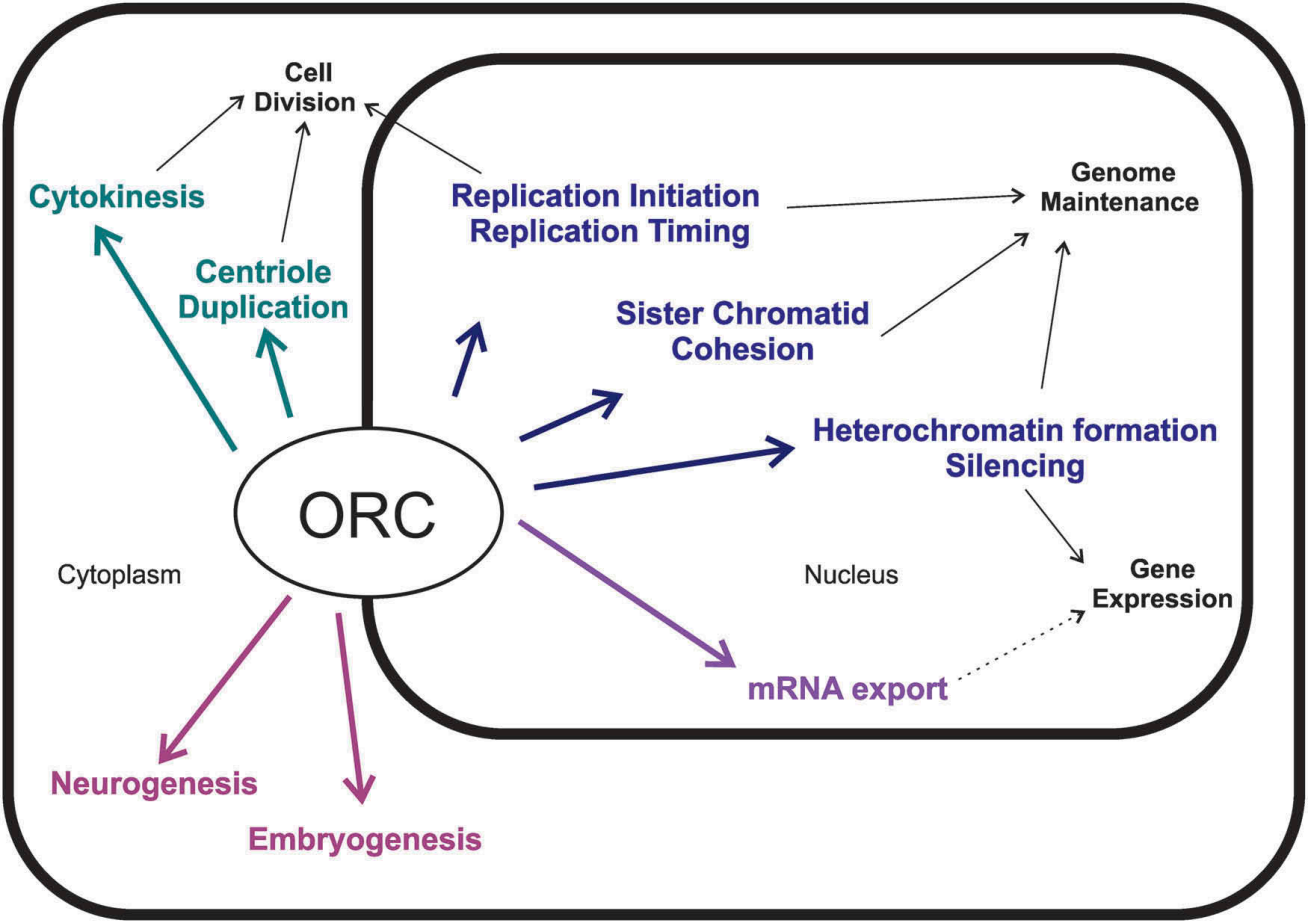
Various biological processes in which ORC subunits are involved. The majority of their functions ORC fulfills in the nucleus, while some of them are associated with cytoplasm.

At the HMR, SIR-independent silencing has been demonstrated as well [31]. In *S. cerevisiae* strains mutant for the SIR proteins, a restoration of silencing was observed when the *SUM1* gene was subsequently mutated [32] (Figure 1a). Similarly to SIR, the mutant SUM1–1 protein is recruited to the chromatin by ORC, where SUM1–1 interacts with the histone deacetylase Hst1 [33]. The substitution of one ORC-dependent silencing mechanism with another highlights the essential role of ORC in the recruitment of repressors to the chromatin.

Initially, during genetic analysis of the yeast mutants, the functions of ORC in genomic replication were recognized as being distinct from its functions in silencing [34]. Later, the independence of the silencing function from the ORC role in replication was confirmed by establishment of silencing on episomes lacking replication origins [35]. Thus, the independence of ORC’s silencing function from replication has been documented, while for the majority of other nonreplicative functions, this question remains open.

In higher eukaryotes, the involvement of ORC in the formation of heterochromatin is related to the interaction with the heterochromatin protein 1 (HP1) [36]. HP1 directly interacts with ORC1 in *Drosophila melanogaster* and *Xenopus laevis* [37]. Moreover, in *Drosophila* mutations in the *Orc2* gene disturb the normal localization of HP1 on heterochromatin [38] and suppress the position effect variegation [37], which is directly dependent on the HP1 function [39]. In *Drosophila*, the ORC-HP1 complex also includes the HP1/ORC-associated protein (HOAP) [40] (Figure 1a). This complex was found on pericentromeric heterochromatin [41]. The proposed model for the functioning of ORC-HP1-HOAP complex is similar to the mechanism of action of the SIR proteins in yeast. ORC, together with HOAP [38,42], recruits HP1 and hystone methyltransferases (HMTs) to the pericentromeric DNA [41,42].

ORC and HP1 co-localization on heterochromatin was also demonstrated in the mammalian cells [43–47]. Nevertheless, it is still unclear whether ORC interact with HP1 on the same regions of heterochomatin, because depletion of the individual ORC subunits in human cells has different outcomes on HP1 localization on heterochromatin. For instance, unlike ORC1 and ORC5, ORC2 and ORC3 participate in HP1 recruiting to the centromeric regions (Figure 1b) [46].

In mammalian cells, there is an additional protein which interacts with the ORC complex and is involved in heterochromatin formation, named the ORC-associated protein (ORCA) also known as Leucine-rich Repeat and WD repeat-containing protein 1 (LRWD1) (Figure 1a). ORCA is associated with ORC, stabilizes ORC on chromatin, and facilitates pre-RC assembly [48].

ORCA-ORC exist in a single complex with histone methyltransferases (HMTs) which perform the heterochromatin-specific histone modification [49–51]. ORCA interacts directly with repressive histone marks, such as H3K9me3, H3K27me3, and H4K20me3 [52] and removal of ORCA from human cells causes aberrations in the chromatin architecture and significantly reduces H3K9 di- and tri-methylation [53]. It is likely that ORCA acts as a scaffold protein that enables the formation of multiple HMT complexes at heterochromatic sites, thereby facilitating the chromatin organization.

Other intriguing observations of the participation of ORC in the formation of heterochromatin were obtained for ORC2. It has been shown that ORC2 localizes to the centromeres during the cell cycle G2/M phase [43,54,55] (Figure 1a). Post-translational modification of ORC2 with the SUMO2 attachment is important for the maintenance of centromeric histone H3 methylation status, and the prevention of heterochromatin re-replication via the recruitment of demethylase KDM5A [8] (Figure 1b). Moreover, the regulation of ORC2 SUMOylation at the G2/M phase is critical for smooth progression of the mitotic cycle of cells. Interestingly, ORC2 interacts with ORCA directly [56] and protects it from ubiquitination and proteasome degradation [56]. Despite this, the role of ORCA in the maintenance of centromeric heterochromatin is not yet clear.

ORC is also involved in the heterochromatin formation at telomeres, via an interaction between ORC1, the TRF2 protein and the telomere-repeat encoding (TERRA) RNA [57,58] (Figure 1c). In particular, TRF2 binds to the telomeric double-stranded repeat, while the TERRA RNA facilitates an interaction between ORC1 and the GAR domain of TRF2. Depletion of the TERRA RNA leads to a disruption of the ternary ORC-TERRA-TRF2 complex and causes a decrease in the HP1 level at telomeres, a loss of the H3K9me3 mark, an increase in telomere disfunction induced foci, and aberrations at the metaphase telomeres (Figure 1c).

Recently, it has been shown that ORC is involved not only in heterochromatin formation, but also in the regulation of transcription of particular genes. Hossain and Stillman demonstrated how the ORC1 protein acts in the repression of the E2F1-dependent transcription of the *CCNE1* gene, which encodes the Cyclin E protein [46,59] (Figure 1a). According to this study, ORC1 binds to the Retinoblastoma (RB) protein at the *CCNE1* promoter and recruits the SUV39H1 histone-methyltransferase, which introduces the H3K9me3 mark, and thus, facilitates HP1 binding. Interestingly, the CDC6 protein plays an opposing role in this process. It binds to the Cyclin E/CDK2 kinase and eliminates the *CCNE1* repression. This study not only uncovered the role of ORC1 in the expression of a single gene, but also highlighted its regulatory function in the cell cycle progression, since Cyclin E is one of the key factors that regulate the cell cycle. Moreover, since ORC1 is known to bind both SUV39H1 and HP1 [46], it may participate in the regulation of transcription of other E2F1-dependent genes during G1 phase.

Despite a large number of facts pointing to the participation of ORC in heterochromatin formation, and the regulation of gene expression, the specific role of the complex in these processes remains unclear. In most studies, it appears that ORC acts as an intermediate participant in a complex chain of protein-protein interactions which lead to heterochromatin formation. ORC either directly or via other proteins interacts with enzymes, such as histone deacetylases and histone methyltransferases, which label nucleosomes with epigenetic marks and facilitate the formation of heterochromatin structure (Figure 1).

Importantly, although in yeast the functions of the ORC in silencing and replication were separated in genetic experiments, in metazoans, such experiments have not yet been conducted. Apparently, in higher eukaryotes the function of heterochromatin formation and maintenance is tightly associated with the function of replication and it is rather difficult to separate them. Thus, it is still not clear, whether ORC is required for maintenance of heterochromatin structure or if ORC interacts with heterochromatin-associated proteins because heterochromatin structure is hard to replicate. This point of view was discussed in a review [60].

Despite the fact that ORC subunits are evolutionarily conserved, in evolving from yeast to metazoans the ORC complex has lost the ability to bind DNA in a sequence-specific manner. Thus, while in yeast there are replication origins of two types: either of high ORC-DNA affinity or of appropriate chromatin environment; in metazoans, the first type of origin was lost; therefore chromatin state plays a major role in this case. Apparently, while in yeast the ORC complex can function as a platform for recruitment of other proteins such as SIR1 (or mutant SUM1–1), in metazoans, ORC requires a platform to which the complex itself can be recruited.

Although it was reported that ORC2 interacts with the repressive chromatin marks H3K9me3 and H3K27me3 in *S. cerevisiae* [61], the subunits of ORC do not have domains for reading post-translational modification of histones attributed to repressive chromatin. This in turn led to the appearance of proteins such as ORCA in mammals or HOAP in *Drosophila*, which directly interact with HMTs and are involved in the formation and maintenance of heterochromatin by binding histone modifications.

At the same time, despite the functional similarity of ORCA and HOAP, these proteins are not homologues. Unlike HOAP, ORCA is tightly associated with the ORC complex and participates in the formation of heterochromatin in centromeric region and at the repressed loci. Besides, HOAP of *Drosophila* was found not only on the centromeres, where it interacted with ORC2, but also on the *Drosophila* telomeres. Moreover, HOAP interacts with many factors, including HipHop [62] and Umbrea [63]. Furthermore, the appearance of such different proteins as ORCA and HOAP indicates that despite the evolutionary conservative core, the ORC complex has been prominently altered during the course of evolution and acquired new properties and new functions.

## Sister chromatid cohesion

Another ORC function is related to chromosomal organization and segregation. As a result of DNA replication during S phase, pairs of sister chromatids are bound to each other. The chromatids remain paired until the transition from the metaphase to anaphase, when the contacts between them break and chromatids are pulled to the opposite poles of the cell [64]. Genetic studies have found that ORC mutations in yeast (*orc2–1* and *orc5–1*) and in *Drosophila* (*orc6)* cause numerous cell cycle defects, including mitotic defects [65,66]. Mutational analyses in yeast revealed interactions between the ORC genes and the genes of proteins responsible for the sister chromatid cohesion. Double mutants showed an enhanced impairment of sister chromatid cohesion, which suggested an involvement of ORC in this process [65]. However, whether ORC plays a direct role in sister chromatid cohesion or the observed effects are the consequence of a replication impairment remained for a long time unclear.

From the start of DNA replication, the establishment and maintenance of the linkage between sister chromatids is carried out by the cohesin complex, which forms a ring encompassing the paired DNA molecules [67]. The recruitment of the cohesin complex components occurs prior to the start of replication, namely, during G1 phase in yeast [68] and telophase in higher eukaryotes [69–72], and coincides with the loading of pre-replication complex onto the chromosome, which indicates that ORC could play a role in cohesin recruitment to the chromatin. Several studies in the yeast *Schizosaccharomyces pombe*, confirmed that the ORC5 interacts with cohesin complex components and is necessary for its recruitment to centromeric regions of chromosomes [73]. It has been demonstrated *in vitro*, that in *X. laevis* the pre-RCs are required for the loading of the cohesin complex onto DNA [74]. Remarkably, the pre-RC facilitates recruitment of not only the cohesin complex itself, but also the acetyltransferase which is required for the cohesin complex function [75]. The authors also showed that the role of the pre-RC in cohesin recruitment is independent from its role in replication, and those pre-RCs which interact with cohesin are unable to act as replication origins [75]. Evidence indicating an involvement of the pre-RC in the cohesin recruitment to the chromatin has been obtained for other organisms as well. *In vitro* experiments in *X. laevis* showed that an extract containing pre-RC provides interaction between the human cohesin complex and DNA, and this interaction may be blocked by inhibitors of the pre-RC assembly [76]. ChIP-seq analysis in *D. melanogaster* showed that the majority of ORC binding sites coincides with the sites of cohesin localization, thus supporting evidence for the involvement of pre-RC in cohesin recruitment to DNA [77].

In *S. cerevisiae*, unlike in the higher eukaryotes, an association between the pre-RC and cohesin loading on chromatin has not been found, perhaps in this case, either the cohesin complex is recruited to DNA autonomously, or this process requires other factors [78]. However, in *S. cerevisiae*, ORC is involved in a cohesin-independent mechanism for the sister chromatid segregation. In budding yeast, ORC2 depletion during the G1 phase (after pre-RC assembly) leads to a disruption of the linkage between sister chromatids in spite of the presence of cohesin complex on chromatin [79]. At the same time, in cohesin mutants, sister chromatid cohesion can be restored by an integration of the sequences recognized by ORC into the genome. These observations provide evidence for an independent activity of ORC and the cohesin complex in this case [79]. The results of chromatin immunoprecipitation with microarray analysis (ChIP-on-chip) in *S. cerevisiae*, revealed that cohesin localizes mostly between the regions of an active transcription, and that transcription complexes may thus restrict cohesin spreading [78,80].

Despite ORC involvement in two different mechanisms providing sister chromatid cohesion in yeast and higher eukaryotes, there is no strong evidence for the participation of ORC in this process in the mammalian cells. Therefore, we can only speculate on the role that ORC may play in the recruitment of cohesin to DNA. Obviously, replication and sister chromatid cohesion are two related processes, which should be coordinated in some way. However, interconnetions between these two processes could be more complex than just a direct interaction or recruitment. Thus, unraveling of these mechanisms will require further investigation.

## Replication timing

Although this review is focused on the nonreplicative functions of ORC, the relationship between ORC and the timing of replication of different DNA regions should be mentioned. It has been demonstrated for *S. cerevisiae* that all replication origins can be divided into two groups based on their replication time: the early-replicating sites and the late-replicating sites [81]. The replication time for a certain origin strongly correlates with its affinity to ORC. For example, the late-replicating origins are characterized by low values of the dissociation constant for ORC, while the early-replicating sites are characterized by high dissociation constant values, the interaction of ORC with the replication site in this case depending to a larger extent on the chromatin environment [81]. Thus, the mode of ORC interaction with the replication origins may affect the DNA replication timing in *S. cerevisiae*.

In metazoan cells, replication starts first at the euchromatic regions, where early-replicating sites are located, while the heterochromatin contains the late-replicating sites and is duplicated later [82]. Obviously, ORC exerts its effects on the replication timing of different chromatin regions. In *D. melanogaster*, a mutation in the *Orc2* gene impairs euchromatic replication, specifically, the time of replication is shifted and euchromatin duplicates after heterochromatin [83]. In this background, replication also remains incomplete at certain regions [83]. This leads to the cell cycle arrest during mitosis, which is accompanied by the abnormal chromosome condensation, chromosome fragmentation, and a partial absence of cohesion between sister chromatids. Similar cell phenotypes were described in *Drosophila* mutants for ORC5, MCM4, and other replication factors [83]. A relationship between the ORCA-ORC complex and replication timing has been detected in human cell culture [50,52]. It was demonstrated that a decrease in the ORCA content in the cell results in several defects in chromosomal organization and causes changes in the replication time of late-replicating regions [52].

Notwithstanding numerous facts indicating an involvement of ORC in replication timing, for a long time it remained unclear, whether ORC indeed participates in the regulation of replication timing. Obviously, the difference in the replication timing of various genome regions is a result of regulation of the firing of potential replication origins, which are supposed to be located as frequently as required to ensure the replication of the entire genome in the S phase. The genome-wide studies of the last decade have provided new data about the distribution of the binding sites for the ORC complex throughout the genome. In the yeast *S. cerevisiae*, ORC recognizes and binds a specific DNA sequence, known as the ACS motif [1]. Although the yeast genome contains about 12,000 matches to the ACS motif, only ~ 220–400 of them are functional and are bound by ORC, apparently because the active replication origins are determined substantially by the local chromatin environment [84].

Interestingly, the number of ORC binding sites increases in accordance with the genome size, which allows the average size of a replicon unit to be evolutionarily constant. According to the latest data, ORC has a large number of binding sites throughout the genome: about 5,000 in *Drosophila* [77] and about 52,000 in mammals [85]. The analysis of the origin activity revealed an existence of more than 80,000 of origins that were active in various human and mouse ESC lines. Furthermore, about 35% of them were shared across several cell lines [86]. Thus, ORC has an excess of potential binding sites, which enables a high plasticity of the replication.

Analysis of the genome-wide data has also revealed several unexpected features of the ORC binding sites in metazoans. In particular, ORC-binding sites coincide with DNAse I hypersensitive sites and histone modifications of active chromatin [87–92]. The majority of the ORC binding sites coincide with transcription regulatory elements, such as promoters and transcriptional enhancers [85]. The preference of ORC to bind to open chromatin also explains a high correlation between the ORC binding sites and the binding sites of various transcription factors, which has been described previously [86,93,94]. This feature of ORC binding to chromatin leads to the fact that, in euchromatin, ORC binding sites are located much closer to each other, and thus more often, than those in heterochromatin. It is likely, that in the heterochromatin, ORC also binds to the islands of ‘locally open’ chromatin. For example, in the heterochromatin of *Drosophila*, ORC binds predominantly to the insulators of the Su(Hw) type [95].

In a recent study [85], mathematical simulations of replication (during the S phase of the cell cycle) have shown that the firing of replication origins is, most likely, a stochastic process. According to this model, frequent distribution of ORC binding sites in the euchromatin results in its frequent firing by chance and, therefore, in the earlier replication of the whole region, compared to heterochromatic regions [85]. This model explains not only the existence of the early- and late-replicating regions, due to the irregular distribution of ORC binding sites throughout the genome, but also their correlation with eu- and heterochromatin.

Recently, it has been shown that the borders of Topologically Associated Domains (TADs) determine the boundaries of the Replication Domains. In particular, the regions of early replication coincide with the TADs of euchromatin, whereas the TADs of heterochromatin contain the late replicating regions and timing transition regions [96]. Thus, the locations of TADs determine the difference between the early- and the late-replicating regions.

The current view is that in mammals the ORC does not regulate replication timing by any particular mechanism. The early and late regions are merely defined by the frequency of the ORC binding and the spatial chromatin folding.

## Centrosome duplication and cytokinesis

In addition to its participation in several processes within the cell nucleus, ORC has a number of cytoplasmic functions, one of which is an involvement of certain subunits of the complex in the regulation of centrosome duplication [18,19].

In metazoan, centrosomes consist of two centrioles and serve as microtubule-organizing centers during cell division. Upon the completion of cell division each of the daughter cells receives only one centrosome which is further duplicated and reorganized during the interphase to be ready for the next round of division [97]. Immunostaining of mammalian cells revealed the presence of the ORC1–4 proteins at centrosomes [98], and demonstrated that through the cell division, ORC2 localizes at the centromeric regions of chromosomes [43,54,55,98]. In mammalian cells, ORC2 depletion leads not only to defects in the DNA replication, but also to abnormalities during cell division, including noticeable defects in chromosome segregation and centrosome division, however, the precise role of ORC in these processes so far remains unclear [43]. The subsequent studies have found that ORC1 contains the centrosomal localization sequence and is directly involved in the regulation of centrosome division, its role being tightly associated with the functions of cyclins [18,19]. The precise mechanism of its action, has not yet been described, although it has been established that ORC1 inhibits the kinase activity of the Cyclin A/CDK2 and Cyclin E/CDK2 complexes through its interaction with cyclins [18,99]. As a result, ORC1 blocks Cyclin E-dependent centriole division, providing for a single round of centrosome duplication. Moreover, it is essential that the activity of the Cyclin E/CDK2 complex is kept inhibited until the early S phase, when Cyclin E is completely degraded, which coincides with ORC1 degradation through the ubiquitin-dependent pathway [47]. It should be noted that the mechanism of ORC involvement in centrosome duplication requires further investigation and the role of the other ORC subunits, which were detected at the centrosomes, still remain obscure.

Another additional cytoplasmic function of ORC is the engagement of the smallest subunit of ORC, ORC6, in the cytokinesis in *Drosophila* and mammals. Immunostaining of cells with antibodies against ORC6 revealed that a considerable portion of this protein is present in the cytoplasm and is localized to the cell membrane and in the cleavage furrow [20,21]. ORC6 depletion in *D. melanogaster* cells results in defects in cytokinesis [66]. The ORC6 protein possesses two functional domains. The conservative N-terminal domain is responsible for DNA interaction, and in metazoans shows structural homology with the transcription factor TFIIB [100]. A small variable domain at the C-terminus is responsible for interactions with other proteins, and with ORC3, in particular [20,101,102]. Besides that, it was demonstrated that the C-terminal domain contains a motif which mediates an ORC6 interaction with the Pnut protein, a component of the septin complex [20]. ORC6 promotes septin polymerization due to its ability to form a dimer [102,103]. Although the molecular mechanism for the septin filament assembly still remains poorly understood, the results obtained so far provide strong evidence for the direct involvement of ORC6 in this process. The role of ORC6 in cytokinesis in *Drosophila* and mammals is another example of accessory functions of the ORC subunits in the processes related to the cell division. Along with the growth of evolutionary complexity of organisms, the ORC complex functions have become more elaborate and new functions of the ORC sub-complexes and individual subunits have emerged.

## RNA-related nonreplicative functions of ORC

One of the most intriguing features of the ORC complex is its ability to interact with RNA. Interestingly, up to now several groups have already demonstrated interactions between ORC and different types of the non-coding RNAs. The ORC complex of *Tetrahymena thermophila* contains an integral 26T RNA subunit that participates in rDNA origin recognition [104]. As mentioned above, the mammalian ORC is recruited to telomeres via its interaction with TERRA RNA [57]. In mammals, the ORC complex interacts with Y RNAs [105], which are involved in DNA replication [106]. It has also been shown that the recruitment of ORC to the Epstein–Barr virus origin is RNA-dependent [107].

These observations are examples of RNA-mediated recruitment of the ORC complex to chromatin. However, recently we have shown, that the ORC complex interacts with coding mRNAs and participates in mRNA export in *D. melanogaster*. Moreover, ORC interacts with TREX-2, the complex of general mRNA nuclear export [17,108]. Besides that, we have shown an interaction between the ORC subunits and the Nxf1 protein, the mRNA transport receptor [17]. Most likely, TREX-2 facilitates ORC binding to mRNP, which, in turn, promotes the Nxf1 recruitment. The RNAi knockdown of ORC subunits results in a dramatic disruption of mRNA export from the nucleus to cytoplasm. Thus, we have demonstrated that ORC plays a key role in mRNA export in *Drosophila* through its interaction with mRNP and its involvement in the Nxf1 recruitment.

All of these studies reveal that ORC is able to bind to different types of RNA and to acquire new functions due to these interactions. Although further research is required to determine the role of the ORC-RNA interaction in various biological processes, this data is very interesting and extends dramatically our understanding of ORC functions. Interestingly, it has recently been shown that human ORC has a much higher affinity to RNA than to either the double-stranded or single-strainded DNA *in vitro* [109], which encourages further investigation of RNA-ORC interactions *in vivo*.

## Neurogenesis and embriogenesis

Among the non-replicating functions of ORC, one of the most unexpected is its role in neurogenesis. Data collected in the study of *Drosophila*, mice, and rats revealed participation of individual ORC subunits in the functioning of the central nervous system in metazoans.

In *Drosophila*, the *latheo* gene, which encodes the ORC3 protein, is expressed in the adult central nervous system [110]. Interestingly, the ORC3 protein localizes in the neuromuscular junctions (NMJ) and is enriched at the synaptic boutons of *Drosophila* larvae. Mutations of *latheo* cause defects in synaptic transmission and plasticity [111]. Functionally, *latheo* mutations reduce memory immediately after training [112]. This points to a specific role which the ORC3 protein plays at the early stages of learning and memory.

In mammals, the ORC2–5 subunits are highly expressed in the adult brain [113]. The cerebellar granular cells grown in culture demonstrate a particular dynamic of *ORC3* expression, i. e., the peak of expression coincides with the period of the highest growth rate of dendrites. Similar results were obtained in the intact cerebellum, where ORC3 was detected in the cytoplasm of Purkinje cells at 6–8 days of prenatal development [114]. In mammalian neuronal cells, ORC3–5 are enriched in membrane fractions and localize to the somatodendritic compartments [22]. Knockout of either ORC3 or ORC5 leads to severe impairments of dendrite growth and branching in the cultured hippocampal neurons. Besides that, the ORC2–5 sub-complex is required for the development of dendritic branches and spine formation [113].

It is therefore apparent that in higher eukaryotes, subunits of the ORC complex (except ORC1) are involved in the regulation of dendritogenesis and synapse formation in the postmitotic neurons. However, the mechanism of the ORC involvement in the neurogenesis is still poorly understood and needs further investigation.

There are also exciting findings about the localization of ORC subunits during early embryogenesis of mammals. Recently, Dr. Ward with colleagues [115] showed that after fertilization, the ORC1, 2, 3, and 5 proteins localize between the separating maternal chromosomes, while the ORC6 subunit is localized to the periphery of the nucleoli at all zygotic stages. This unexpected distribution of ORC proteins suggest their potential roles in mitosis and chromatin segregation in the mammalian zygote [115].

At the same time, it was found that during both female meiotic divisions ORC4 surrounds the chromatin that will be discarded into the polar body and then binds to the chromosomes at the anaphase. This finding suggests that ORC4 may be engaged in polar body formation [116,117].

## Conclusions

The data accumulated thus far make it obvious that functions of the ORC complex are not restricted to its role in DNA replication, but involve participation in a large number of various biological processes. On the one hand, several ORC functions, such as replication, centrosome duplication, and cytokinesis are related to cell division (Figure 2). The participation of ORC subunits in these processes fulfills the regulatory function and allows it to synchronize all the processes necessary for cell division. On the other hand, there is a group of ORC functions which includes heterochromatin formation, silencing, sister chromatids cohesion, and mRNA export, which are related to the processes occurring in the nucleus and are mostly based on the interaction between ORC and chromatin. These processes relate directly to genome maintenance and gene expression regulation. There are also numerous intriguing reports on the role of ORC in the neurogenesis though the molecular mechanisms underlying this process are to be revealed.

It should be noted that the different functions of ORC are often related to different subunits. The most intricate example is ORC1. ORC1 is a crucial protein for pre-RC formation. The cellular level of the ORC1 protein is tightly regulated during the cell cycle: ORC1 undergoes degradation right after the G1/S transition and the protein level is restored by the beginning of G2. This system is an additional guarantee of re-replication prevention. Moreover, ORC1 is engaged in the process of centrosome division in the cytoplasm by the blocking of Cyclin E-dependent centriole division. In G1 phase, ORC1 participates in the regulation of Cyclin E gene expression, providing global regulation of the cell cycle. Thus, ORC1 coordinates numerous processes required for correct cell cycle progression in various compartments of the cell. Such an elaborate role of only one subunit of the ORC complex suggests a possible existence of new and unexpected additional functions of ORC related to the cell cycle.

Although ORC1 is degraded after G1/S transition and the rest of the ORC complex is stable during the entire cell cycle, many functions were discovered for individual ORC subunits. The simultaneous inspection of all ORC subunits encounters technical obstacles, so in many studies the presence of one or more subunits suggests the presence of the whole complex. For example, despite the fact that knockdown of ORC1, ORC2, ORC3 and ORC5 influences the distribution of HP1 differently, it is obvious, that whole complex is involved in this interaction. In some cases, individual ORC subunits act in quite specific way, in the absence of other complex members. For instance, only ORC4 participates in polar body formation of mammalian zygote, and ORC6 alone regulates the cytokinesis in *Drosophila* and mammals.

These observations raise another question, namely how assembly of the ORC complex occurs and how the incorporation of different ORC subunits is regulated. Thus, unraveling of these mechanisms is a goal of further investigation. Therefore, it may be concluded that ORC participates in many processes in the cell and serves as an instrument of regulation and coordination of the cell’s holistic function.
